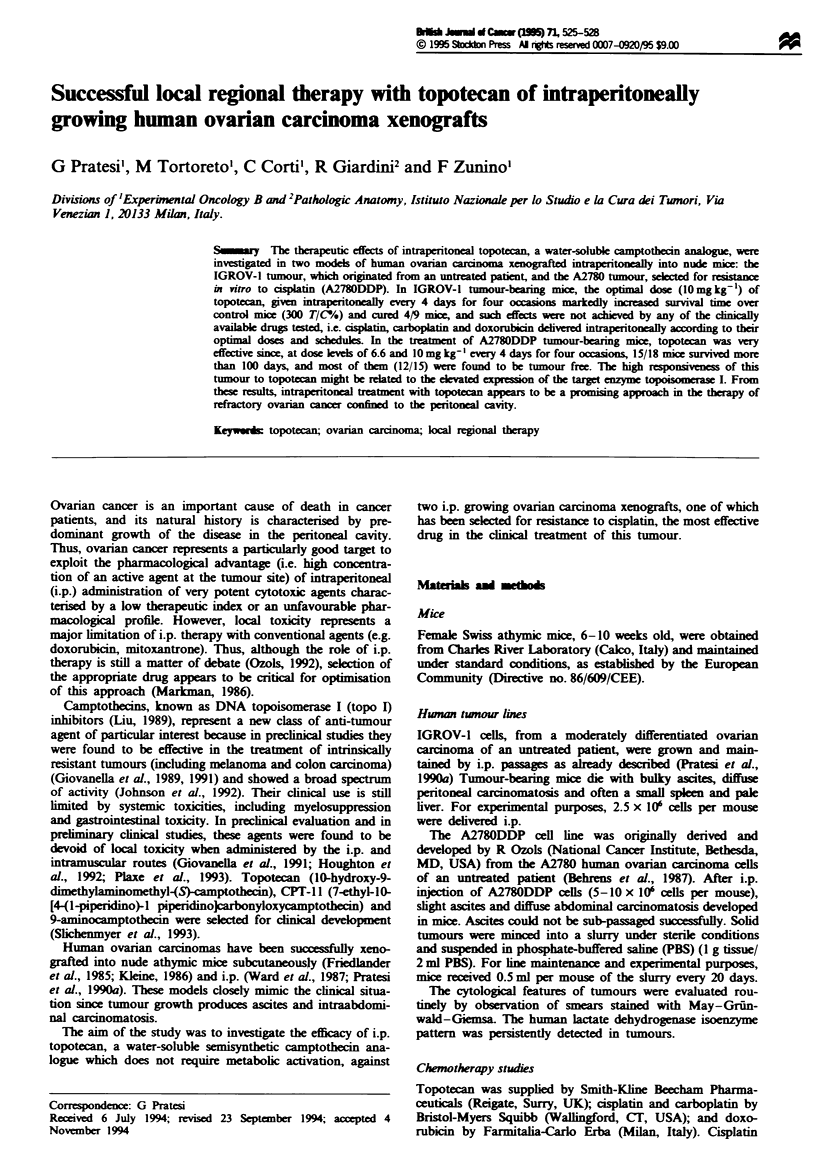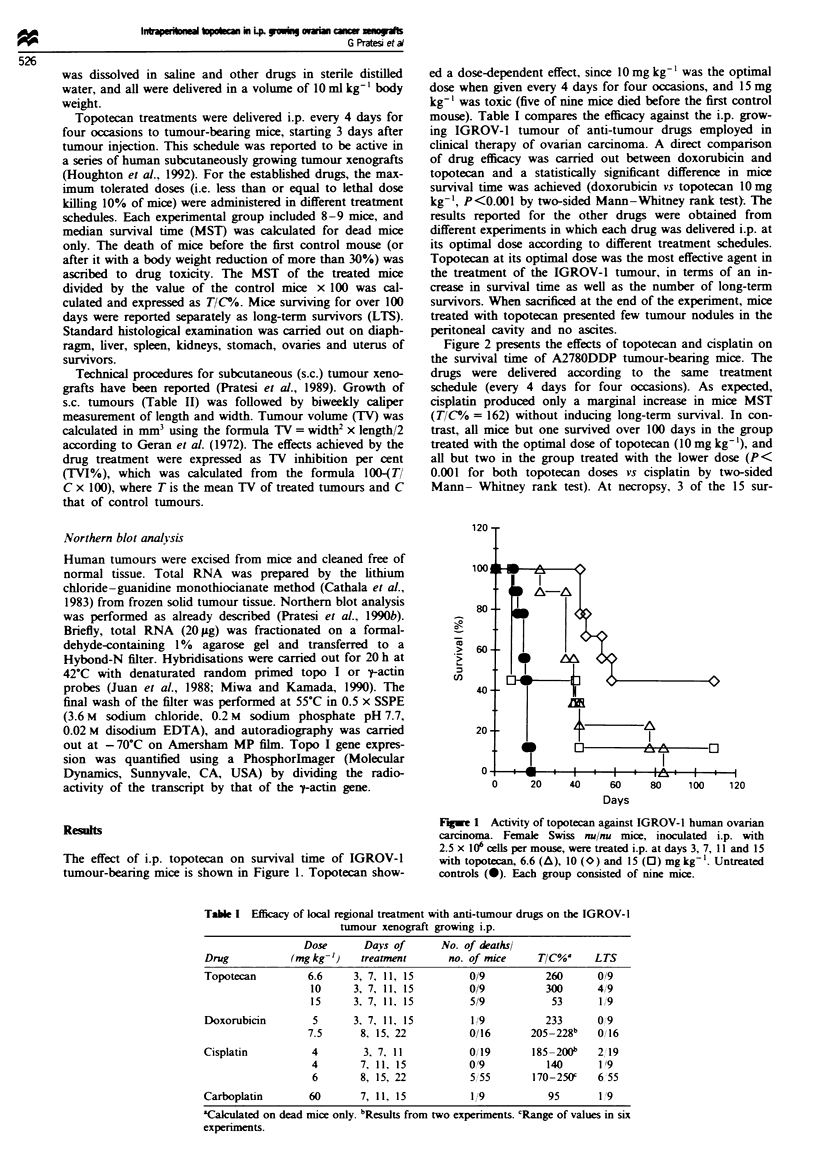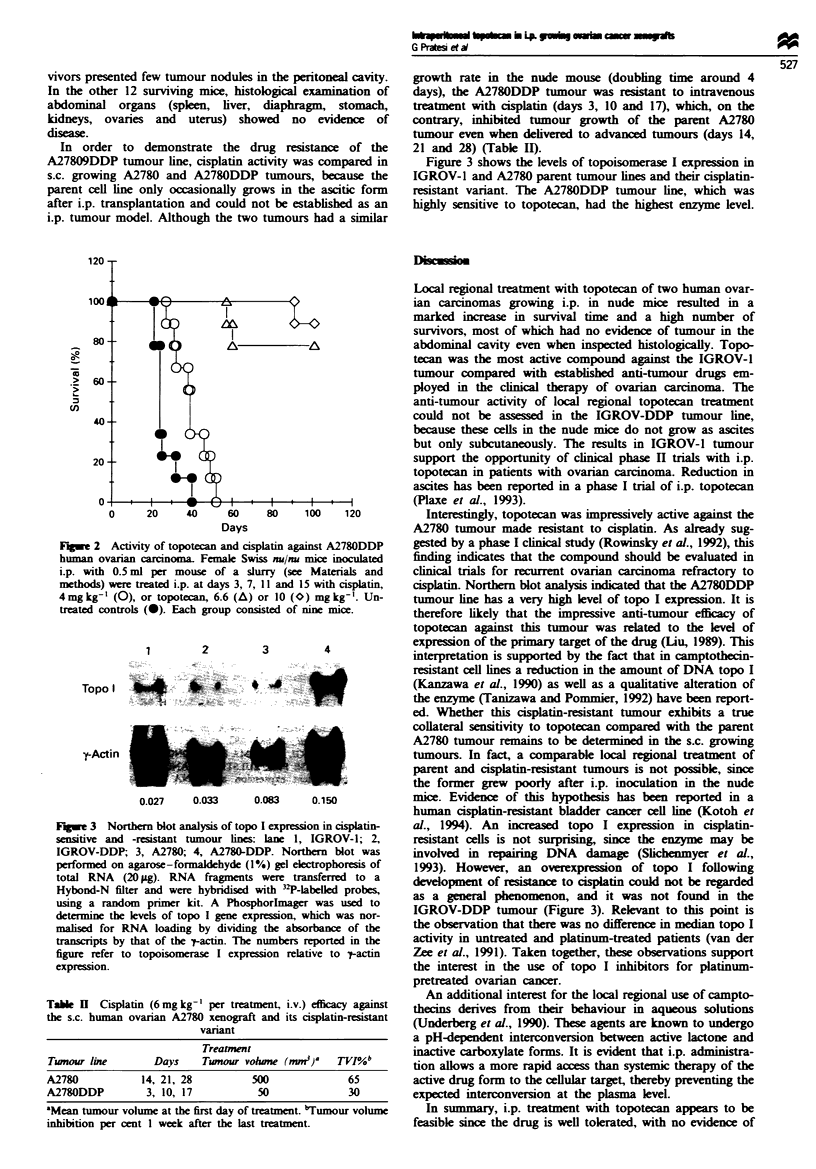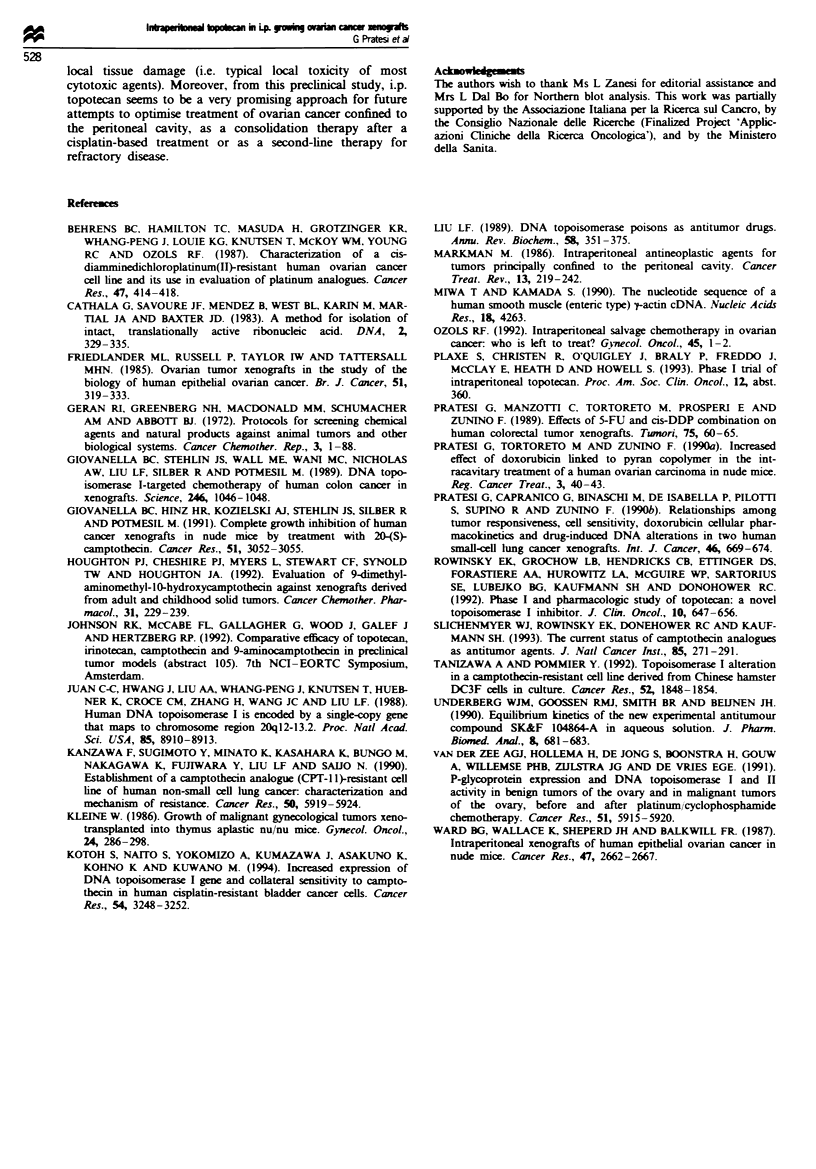# Successful local regional therapy with topotecan of intraperitoneally growing human ovarian carcinoma xenografts.

**DOI:** 10.1038/bjc.1995.104

**Published:** 1995-03

**Authors:** G. Pratesi, M. Tortoreto, C. Corti, R. Giardini, F. Zunino

**Affiliations:** Division of Experimental Oncology B, Istituto Nazionale per lo Studio e la Cura dei Tumori, Milan, Italy.

## Abstract

**Images:**


© 1995 Stockton Press All rights reserved 0007-0920/95 $9.00

### Successful local regional therapy with topotecan of intraperitoneally growing human ovarian carcinoma xenografts

G Pratesi[1], M Tortoreto[1], C Corti[1], R Giardini[2] and F Zunino[1]

*Divisions of [1]Experimental Oncology B and [2]Pathologic Anatomy, Istituto Nazionale per lo Studio e la Cura dei Tumori, Via Venezian 1, 20133 Milan, Italy.*

**Summary** The therapeutic effects of intraperitoneal topotecan, a water-soluble camptothecin analogue, were investigated in two models of human ovarian carcinoma xenografted intraperitoneally into nude mice: the IGROV-1 tumour, which originated from an untreated patient, and the A2780 tumour, selected for resistance *in vitro* to cisplatin (A2780DDP). In IGROV-1 tumour-bearing mice, the optimal dose (10 mg $kg^{-1}$) of topotecan, given intraperitoneally every 4 days for four occasions markedly increased survival time over control mice (300 *T/C*%) and cured 4/9 mice, and such effects were not achieved by any of the clinically available drugs tested, i.e. cisplatin, carboplatin and doxorubicin delivered intraperitoneally according to their optimal doses and schedules. In the treatment of A2780DDP tumour-bearing mice, topotecan was very effective since, at dose levels of 6.6 and 10 mg $kg^{-1}$ every 4 days for four occasions, 15/18 mice survived more than 100 days, and most of them (12/15) were found to be tumour free. The high responsiveness of this tumour to topotecan might be related to the elevated expression of the target enzyme topoisomerase I. From these results, intraperitoneal treatment with topotecan appears to be a promising approach in the therapy of refractory ovarian cancer confined to the peritoneal cavity.

**Keywords:** topotecan; ovarian carcinoma; local regional therapy

Ovarian cancer is an important cause of death in cancer patients, and its natural history is characterised by predominant growth of the disease in the peritoneal cavity. Thus, ovarian cancer represents a particularly good target to exploit the pharmacological advantage (i.e. high concentration of an active agent at the tumour site) of intraperitoneal (i.p.) administration of very potent cytotoxic agents characterised by a low therapeutic index or an unfavourable pharmacological profile. However, local toxicity represents a major limitation of i.p. therapy with conventional agents (e.g. doxorubicin, mitoxantrone). Thus, although the role of i.p. therapy is still a matter of debate (Ozols, 1992), selection of the appropriate drug appears to be critical for optimisation of this approach (Markman, 1986).

Camptothecins, known as DNA topoisomerase I (topo I) inhibitors (Liu, 1989), represent a new class of anti-tumour agent of particular interest because in preclinical studies they were found to be effective in the treatment of intrinsically resistant tumours (including melanoma and colon carcinoma) (Giovanella *et al.*, 1989, 1991) and showed a broad spectrum of activity (Johnson *et al.*, 1992). Their clinical use is still limited by systemic toxicities, including myelosuppression and gastrointestinal toxicity. In preclinical evaluation and in preliminary clinical studies, these agents were found to be devoid of local toxicity when administered by the i.p. and intramuscular routes (Giovanella *et al.*, 1991; Houghton *et al.*, 1992; Plaxe *et al.*, 1993). Topotecan (10-hydroxy-9-dimethylaminomethyl-(*S*)-camptothecin), CPT-11 (7-ethyl-10-[4-(1-piperidino)-1 piperidino]carbonyloxycamptothecin) and 9-aminocamptothecin were selected for clinical development (Slichenmyer *et al.*, 1993).

Human ovarian carcinomas have been successfully xenografted into nude athymic mice subcutaneously (Friedlander *et al.*, 1985; Kleine, 1986) and i.p. (Ward *et al.*, 1987; Pratesi *et al.*, 1990*a*). These models closely mimic the clinical situation since tumour growth produces ascites and intraabdominal carcinomatosis.

The aim of the study was to investigate the efficacy of i.p. topotecan, a water-soluble semisynthetic camptothecin analogue which does not require metabolic activation, against two i.p. growing ovarian carcinoma xenografts, one of which has been selected for resistance to cisplatin, the most effective drug in the clinical treatment of this tumour.

### Materials and methods

### *Mice*

Female Swiss athymic mice, 6-10 weeks old, were obtained from Charles River Laboratory (Calco, Italy) and maintained under standard conditions, as established by the European Community (Directive no. 86/609/CEE).

### *Human tumour lines*

IGROV-1 cells, from a moderately differentiated ovarian carcinoma of an untreated patient, were grown and maintained by i.p. passages as already described (Pratesi *et al.*, 1990*a*) Tumour-bearing mice die with bulky ascites, diffuse peritoneal carcinomatosis and often a small spleen and pale liver. For experimental purposes, $2.5 \times 10^6$ cells per mouse were delivered i.p.

The A2780DDP cell line was originally derived and developed by R Ozols (National Cancer Institute, Bethesda, MD, USA) from the A2780 human ovarian carcinoma cells of an untreated patient (Behrens *et al.*, 1987). After i.p. injection of A2780DDP cells ($5-10 \times 10^6$ cells per mouse), slight ascites and diffuse abdominal carcinomatosis developed in mice. Ascites could not be sub-passaged successfully. Solid tumours were minced into a slurry under sterile conditions and suspended in phosphate-buffered saline (PBS) (1 g tissue/2 ml PBS). For line maintenance and experimental purposes, mice received 0.5 ml per mouse of the slurry every 20 days.

The cytological features of tumours were evaluated routinely by observation of smears stained with May-Grünwald-Giemsa. The human lactate dehydrogenase isoenzyme pattern was persistently detected in tumours.

### *Chemotherapy studies*

Topotecan was supplied by Smith-Kline Beecham Pharmaceuticals (Reigate, Surry, UK); cisplatin and carboplatin by Bristol-Myers Squibb (Wallingford, CT, USA); and doxorubicin by Farmitalia-Carlo Erba (Milan, Italy). Cisplatin

Correspondence: G Pratesi
Received 6 July 1994; revised 23 September 1994; accepted 4 November 1994

was dissolved in saline and other drugs in sterile distilled water, and all were delivered in a volume of 10 ml $kg^{-1}$ body weight.

Topotecan treatments were delivered i.p. every 4 days for four occasions to tumour-bearing mice, starting 3 days after tumour injection. This schedule was reported to be active in a series of human subcutaneously growing tumour xenografts (Houghton *et al.*, 1992). For the established drugs, the maximum tolerated doses (i.e. less than or equal to lethal dose killing 10% of mice) were administered in different treatment schedules. Each experimental group included 8–9 mice, and median survival time (MST) was calculated for dead mice only. The death of mice before the first control mouse (or after it with a body weight reduction of more than 30%) was ascribed to drug toxicity. The MST of the treated mice divided by the value of the control mice × 100 was calculated and expressed as *T*/*C*%. Mice surviving for over 100 days were reported separately as long-term survivors (LTS). Standard histological examination was carried out on diaphragm, liver, spleen, kidneys, stomach, ovaries and uterus of survivors.

Technical procedures for subcutaneous (s.c.) tumour xenografts have been reported (Pratesi *et al.*, 1989). Growth of s.c. tumours (Table II) was followed by biweekly caliper measurement of length and width. Tumour volume (TV) was calculated in $mm^3$ using the formula $TV = width^2 \times length/2$ according to Geran *et al.* (1972). The effects achieved by the drug treatment were expressed as TV inhibition per cent (TVI%), which was calculated from the formula $100-(T/C \times 100)$, where *T* is the mean TV of treated tumours and *C* that of control tumours.

*Northern blot analysis*

Human tumours were excised from mice and cleaned free of normal tissue. Total RNA was prepared by the lithium chloride–guanidine monothiocianate method (Cathala *et al.*, 1983) from frozen solid tumour tissue. Northern blot analysis was performed as already described (Pratesi *et al.*, 1990*b*). Briefly, total RNA (20 μg) was fractionated on a formaldehyde-containing 1% agarose gel and transferred to a Hybond-N filter. Hybridisations were carried out for 20 h at 42°C with denaturated random primed topo I or γ-actin probes (Juan *et al.*, 1988; Miwa and Kamada, 1990). The final wash of the filter was performed at 55°C in 0.5 × SSPE (3.6 M sodium chloride, 0.2 M sodium phosphate pH 7.7, 0.02 M disodium EDTA), and autoradiography was carried out at −70°C on Amersham MP film. Topo I gene expression was quantified using a PhosphorImager (Molecular Dynamics, Sunnyvale, CA, USA) by dividing the radioactivity of the transcript by that of the γ-actin gene.

### Results

The effect of i.p. topotecan on survival time of IGROV-1 tumour-bearing mice is shown in Figure 1. Topotecan showed a dose-dependent effect, since 10 mg $kg^{-1}$ was the optimal dose when given every 4 days for four occasions, and 15 mg $kg^{-1}$ was toxic (five of nine mice died before the first control mouse). Table I compares the efficacy against the i.p. growing IGROV-1 tumour of anti-tumour drugs employed in clinical therapy of ovarian carcinoma. A direct comparison of drug efficacy was carried out between doxorubicin and topotecan and a statistically significant difference in mice survival time was achieved (doxorubicin *vs* topotecan 10 mg $kg^{-1}$, $P<0.001$ by two-sided Mann–Whitney rank test). The results reported for the other drugs were obtained from different experiments in which each drug was delivered i.p. at its optimal dose according to different treatment schedules. Topotecan at its optimal dose was the most effective agent in the treatment of the IGROV-1 tumour, in terms of an increase in survival time as well as the number of long-term survivors. When sacrificed at the end of the experiment, mice treated with topotecan presented few tumour nodules in the peritoneal cavity and no ascites.

Figure 2 presents the effects of topotecan and cisplatin on the survival time of A2780DDP tumour-bearing mice. The drugs were delivered according to the same treatment schedule (every 4 days for four occasions). As expected, cisplatin produced only a marginal increase in mice MST ($T/C\% = 162$) without inducing long-term survival. In contrast, all mice but one survived over 100 days in the group treated with the optimal dose of topotecan (10 mg $kg^{-1}$), and all but two in the group treated with the lower dose ($P<0.001$ for both topotecan doses *vs* cisplatin by two-sided Mann– Whitney rank test). At necropsy, 3 of the 15 sur-

**Figure 1** Activity of topotecan against IGROV-1 human ovarian carcinoma. Female Swiss *nu*/*nu* mice, inoculated i.p. with $2.5 \times 10^6$ cells per mouse, were treated i.p. at days 3, 7, 11 and 15 with topotecan, 6.6 (△), 10 (◇) and 15 (□) mg $kg^{-1}$. Untreated controls (●). Each group consisted of nine mice.

**Table I** Efficacy of local regional treatment with anti-tumour drugs on the IGROV-1 tumour xenograft growing i.p.

| *Drug* | *Dose (mg $kg^{-1}$)* | *Days of treatment* | *No. of deaths/ no. of mice* | *T/C%*[a] | *LTS* |
|---|---|---|---|---|---|
| Topotecan | 6.6 | 3, 7, 11, 15 | 0/9 | 260 | 0/9 |
| | 10 | 3, 7, 11, 15 | 0/9 | 300 | 4/9 |
| | 15 | 3, 7, 11, 15 | 5/9 | 53 | 1/9 |
| Doxorubicin | 5 | 3, 7, 11, 15 | 1/9 | 233 | 0/9 |
| | 7.5 | 8, 15, 22 | 0/16 | 205–228[b] | 0/16 |
| Cisplatin | 4 | 3, 7, 11 | 0/19 | 185–200[b] | 2/19 |
| | 4 | 7, 11, 15 | 0/9 | 140 | 1/9 |
| | 6 | 8, 15, 22 | 5/55 | 170–250[c] | 6/55 |
| Carboplatin | 60 | 7, 11, 15 | 1/9 | 95 | 1/9 |

[a]Calculated on dead mice only. [b]Results from two experiments. [c]Range of values in six experiments.

vivors presented few tumour nodules in the peritoneal cavity. In the other 12 surviving mice, histological examination of abdominal organs (spleen, liver, diaphragm, stomach, kidneys, ovaries and uterus) showed no evidence of disease.

In order to demonstrate the drug resistance of the A27809DDP tumour line, cisplatin activity was compared in s.c. growing A2780 and A2780DDP tumours, because the parent cell line only occasionally grows in the ascitic form after i.p. transplantation and could not be established as an i.p. tumour model. Although the two tumours had a similar growth rate in the nude mouse (doubling time around 4 days), the A2780DDP tumour was resistant to intravenous treatment with cisplatin (days 3, 10 and 17), which, on the contrary, inhibited tumour growth of the parent A2780 tumour even when delivered to advanced tumours (days 14, 21 and 28) (Table II).

Figure 3 shows the levels of topoisomerase I expression in IGROV-1 and A2780 parent tumour lines and their cisplatin-resistant variant. The A2780DDP tumour line, which was highly sensitive to topotecan, had the highest enzyme level.

**Figure 2** Activity of topotecan and cisplatin against A2780DDP human ovarian carcinoma. Female Swiss *nu*/*nu* mice inoculated i.p. with 0.5 ml per mouse of a slurry (see Materials and methods) were treated i.p. at days 3, 7, 11 and 15 with cisplatin, 4 mg kg$^{-1}$ (○), or topotecan, 6.6 (△) or 10 (◇) mg kg$^{-1}$. Untreated controls (●). Each group consisted of nine mice.

**Figure 3** Northern blot analysis of topo I expression in cisplatin-sensitive and -resistant tumour lines: lane 1, IGROV-1; 2, IGROV-DDP; 3, A2780; 4, A2780-DDP. Northern blot was performed on agarose–formaldehyde (1%) gel electrophoresis of total RNA (20 μg). RNA fragments were transferred to a Hybond-N filter and were hybridised with $^{32}$P-labelled probes, using a random primer kit. A PhosphorImager was used to determine the levels of topo I gene expression, which was normalised for RNA loading by dividing the absorbance of the transcripts by that of the γ-actin. The numbers reported in the figure refer to topoisomerase I expression relative to γ-actin expression.

**Table II** Cisplatin (6 mg kg$^{-1}$ per treatment, i.v.) efficacy against the s.c. human ovarian A2780 xenograft and its cisplatin-resistant variant

| | Treatment | | |
|---|---|---|---|
| *Tumour line* | *Days* | *Tumour volume (mm$^3$)$^a$* | *TVI%$^b$* |
| A2780 | 14, 21, 28 | 500 | 65 |
| A2780DDP | 3, 10, 17 | 50 | 30 |

$^a$Mean tumour volume at the first day of treatment. $^b$Tumour volume inhibition per cent 1 week after the last treatment.

### Discussion

Local regional treatment with topotecan of two human ovarian carcinomas growing i.p. in nude mice resulted in a marked increase in survival time and a high number of survivors, most of which had no evidence of tumour in the abdominal cavity even when inspected histologically. Topotecan was the most active compound against the IGROV-1 tumour compared with established anti-tumour drugs employed in the clinical therapy of ovarian carcinoma. The anti-tumour activity of local regional topotecan treatment could not be assessed in the IGROV-DDP tumour line, because these cells in the nude mice do not grow as ascites but only subcutaneously. The results in IGROV-1 tumour support the opportunity of clinical phase II trials with i.p. topotecan in patients with ovarian carcinoma. Reduction in ascites has been reported in a phase I trial of i.p. topotecan (Plaxe *et al*., 1993).

Interestingly, topotecan was impressively active against the A2780 tumour made resistant to cisplatin. As already suggested by a phase I clinical study (Rowinsky *et al*., 1992), this finding indicates that the compound should be evaluated in clinical trials for recurrent ovarian carcinoma refractory to cisplatin. Northern blot analysis indicated that the A2780DDP tumour line has a very high level of topo I expression. It is therefore likely that the impressive anti-tumour efficacy of topotecan against this tumour was related to the level of expression of the primary target of the drug (Liu, 1989). This interpretation is supported by the fact that in camptothecin-resistant cell lines a reduction in the amount of DNA topo I (Kanzawa *et al*., 1990) as well as a qualitative alteration of the enzyme (Tanizawa and Pommier, 1992) have been reported. Whether this cisplatin-resistant tumour exhibits a true collateral sensitivity to topotecan compared with the parent A2780 tumour remains to be determined in the s.c. growing tumours. In fact, a comparable local regional treatment of parent and cisplatin-resistant tumours is not possible, since the former grew poorly after i.p. inoculation in the nude mice. Evidence of this hypothesis has been reported in a human cisplatin-resistant bladder cancer cell line (Kotoh *et al*., 1994). An increased topo I expression in cisplatin-resistant cells is not surprising, since the enzyme may be involved in repairing DNA damage (Slichenmyer *et al*., 1993). However, an overexpression of topo I following development of resistance to cisplatin could not be regarded as a general phenomenon, and it was not found in the IGROV-DDP tumour (Figure 3). Relevant to this point is the observation that there was no difference in median topo I activity in untreated and platinum-treated patients (van der Zee *et al*., 1991). Taken together, these observations support the interest in the use of topo I inhibitors for platinum-pretreated ovarian cancer.

An additional interest for the local regional use of camptothecins derives from their behaviour in aqueous solutions (Underberg *et al*., 1990). These agents are known to undergo a pH-dependent interconversion between active lactone and inactive carboxylate forms. It is evident that i.p. administration allows a more rapid access than systemic therapy of the active drug form to the cellular target, thereby preventing the expected interconversion at the plasma level.

In summary, i.p. treatment with topotecan appears to be feasible since the drug is well tolerated, with no evidence of local tissue damage (i.e. typical local toxicity of most cytotoxic agents). Moreover, from this preclinical study, i.p. topotecan seems to be a very promising approach for future attempts to optimise treatment of ovarian cancer confined to the peritoneal cavity, as a consolidation therapy after a cisplatin-based treatment or as a second-line therapy for refractory disease.

**Acknowledgements**

The authors wish to thank Ms L Zanesi for editorial assistance and Mrs L Dal Bo for Northern blot analysis. This work was partially supported by the Associazione Italiana per la Ricerca sul Cancro, by the Consiglio Nazionale delle Ricerche (Finalized Project 'Applicazioni Cliniche della Ricerca Oncologica'), and by the Ministero della Sanita.

**References**

BEHRENS BC, HAMILTON TC, MASUDA H, GROTZINGER KR, WHANG-PENG J, LOUIE KG, KNUTSEN T, McKOY WM, YOUNG RC AND OZOLS RF. (1987). Characterization of a cis-diamminedichloroplatinum(II)-resistant human ovarian cancer cell line and its use in evaluation of platinum analogues. *Cancer Res.*, **47**, 414-418.

CATHALA G, SAVOURE JF, MENDEZ B, WEST BL, KARIN M, MARTIAL JA AND BAXTER JD. (1983). A method for isolation of intact, translationally active ribonucleic acid. *DNA*, **2**, 329-335.

FRIEDLANDER ML, RUSSELL P, TAYLOR IW AND TATTERSALL MHN. (1985). Ovarian tumor xenografts in the study of the biology of human epithelial ovarian cancer. *Br. J. Cancer*, **51**, 319-333.

GERAN RI, GREENBERG NH, MACDONALD MM, SCHUMACHER AM AND ABBOTT BJ. (1972). Protocols for screening chemical agents and natural products against animal tumors and other biological systems. *Cancer Chemother. Rep.*, **3**, 1-88.

GIOVANELLA BC, STEHLIN JS, WALL ME, WANI MC, NICHOLAS AW, LIU LF, SILBER R AND POTMESIL M. (1989). DNA topoisomerase I-targeted chemotherapy of human colon cancer in xenografts. *Science*, **246**, 1046-1048.

GIOVANELLA BC, HINZ HR, KOZIELSKI AJ, STEHLIN JS, SILBER R AND POTMESIL M. (1991). Complete growth inhibition of human cancer xenografts in nude mice by treatment with 20-(S)-camptothecin. *Cancer Res.*, **51**, 3052-3055.

HOUGHTON PJ, CHESHIRE PJ, MYERS L, STEWART CF, SYNOLD TW AND HOUGHTON JA. (1992). Evaluation of 9-dimethyl-aminomethyl-10-hydroxycamptothecin against xenografts derived from adult and childhood solid tumors. *Cancer Chemother. Pharmacol.*, **31**, 229-239.

JOHNSON RK, McCABE FL, GALLAGHER G, WOOD J, GALEF J AND HERTZBERG RP. (1992). Comparative efficacy of topotecan, irinotecan, camptothecin and 9-aminocamptothecin in preclinical tumor models (abstract 105). 7th NCI-EORTC Symposium, Amsterdam.

JUAN C-C, HWANG J, LIU AA, WHANG-PENG J, KNUTSEN T, HUEBNER K, CROCE CM, ZHANG H, WANG JC AND LIU LF. (1988). Human DNA topoisomerase I is encoded by a single-copy gene that maps to chromosome region 20q12-13.2. *Proc. Natl Acad. Sci. USA*, **85**, 8910-8913.

KANZAWA F, SUGIMOTO Y, MINATO K, KASAHARA K, BUNGO M, NAKAGAWA K, FUJIWARA Y, LIU LF AND SAIJO N. (1990). Establishment of a camptothecin analogue (CPT-11)-resistant cell line of human non-small cell lung cancer: characterization and mechanism of resistance. *Cancer Res.*, **50**, 5919-5924.

KLEINE W. (1986). Growth of malignant gynecological tumors xenotransplanted into thymus aplastic nu/nu mice. *Gynecol. Oncol.*, **24**, 286-298.

KOTOH S, NAITO S, YOKOMIZO A, KUMAZAWA J, ASAKUNO K, KOHNO K AND KUWANO M. (1994). Increased expression of DNA topoisomerase I gene and collateral sensitivity to camptothecin in human cisplatin-resistant bladder cancer cells. *Cancer Res.*, **54**, 3248-3252.

LIU LF. (1989). DNA topoisomerase poisons as antitumor drugs. *Annu. Rev. Biochem.*, **58**, 351-375.

MARKMAN M. (1986). Intraperitoneal antineoplastic agents for tumors principally confined to the peritoneal cavity. *Cancer Treat. Rev.*, **13**, 219-242.

MIWA T AND KAMADA S. (1990). The nucleotide sequence of a human smooth muscle (enteric type) γ-actin cDNA. *Nucleic Acids Res.*, **18**, 4263.

OZOLS RF. (1992). Intraperitoneal salvage chemotherapy in ovarian cancer: who is left to treat? *Gynecol. Oncol.*, **45**, 1-2.

PLAXE S, CHRISTEN R, O'QUIGLEY J, BRALY P, FREDDO J, McCLAY E, HEATH D AND HOWELL S. (1993). Phase I trial of intraperitoneal topotecan. *Proc. Am. Soc. Clin. Oncol.*, **12**, abst. 360.

PRATESI G, MANZOTTI C, TORTORETO M, PROSPERI E AND ZUNINO F. (1989). Effects of 5-FU and cis-DDP combination on human colorectal tumor xenografts. *Tumori*, **75**, 60-65.

PRATESI G, TORTORETO M AND ZUNINO F. (1990*a*). Increased effect of doxorubicin linked to pyran copolymer in the intracavitary treatment of a human ovarian carcinoma in nude mice. *Reg. Cancer Treat.*, **3**, 40-43.

PRATESI G, CAPRANICO G, BINASCHI M, DE ISABELLA P, PILOTTI S, SUPINO R AND ZUNINO F. (1990*b*). Relationships among tumor responsiveness, cell sensitivity, doxorubicin cellular pharmacokinetics and drug-induced DNA alterations in two human small-cell lung cancer xenografts. *Int. J. Cancer*, **46**, 669-674.

ROWINSKY EK, GROCHOW LB, HENDRICKS CB, ETTINGER DS, FORASTIERE AA, HUROWITZ LA, McGUIRE WP, SARTORIUS SE, LUBEJKO BG, KAUFMANN SH AND DONOHOWER RC. (1992). Phase I and pharmacologic study of topotecan: a novel topoisomerase I inhibitor. *J. Clin. Oncol.*, **10**, 647-656.

SLICHENMYER WJ, ROWINSKY EK, DONEHOWER RC AND KAUFMANN SH. (1993). The current status of camptothecin analogues as antitumor agents. *J. Natl Cancer Inst.*, **85**, 271-291.

TANIZAWA A AND POMMIER Y. (1992). Topoisomerase I alteration in a camptothecin-resistant cell line derived from Chinese hamster DC3F cells in culture. *Cancer Res.*, **52**, 1848-1854.

UNDERBERG WJM, GOOSSEN RMJ, SMITH BR AND BEIJNEN JH. (1990). Equilibrium kinetics of the new experimental antitumour compound SK&F 104864-A in aqueous solution. *J. Pharm. Biomed. Anal.*, **8**, 681-683.

VAN DER ZEE AGJ, HOLLEMA H, DE JONG S, BOONSTRA H, GOUW A, WILLEMSE PHB, ZIJLSTRA JG AND DE VRIES EGE. (1991). P-glycoprotein expression and DNA topoisomerase I and II activity in benign tumors of the ovary and in malignant tumors of the ovary, before and after platinum/cyclophosphamide chemotherapy. *Cancer Res.*, **51**, 5915-5920.

WARD BG, WALLACE K, SHEPERD JH AND BALKWILL FR. (1987). Intraperitoneal xenografts of human epithelial ovarian cancer in nude mice. *Cancer Res.*, **47**, 2662-2667.